# Age- and Genotype-Associated Specific Expression of IL-1 and TNF Receptors on Immunocompetent Cells

**DOI:** 10.3390/ijms27020807

**Published:** 2026-01-13

**Authors:** Julia Zhukova, Julia Lopatnikova, Filipp Vasilyev, Alina Alshevskaya, Darya Lipa, Sergey Sennikov

**Affiliations:** 1 Federal State Budgetary Scientific Institution Research Institute of Fundamental and Clinical Immunology, Novosibirsk 630099, Russia; lopatnikova18@yandex.ru (J.L.); vasilyevmd@gmail.com (F.V.); dasha.lipa04@gmail.com (D.L.); 2 Federal State Autonomous Educational Institution of Higher Education, I.M. Sechenov First Moscow State Medical University of the Ministry of Health of the Russian Federation (Sechenov University), Moscow 119048, Russia; alkkina@yandex.ru; 3 Institute of Medicine, Ammosov North-Eastern Federal University in Yakutsk, Yakutsk 677013, Russia

**Keywords:** inflammaging, receptors, polymorphisms, genotype, immunocompetent cells

## Abstract

Aging is accompanied by a chronic, low-grade inflammatory state known as “inflammaging,” largely driven by dysregulated signaling of pro-inflammatory cytokines like IL-1 and TNF-α. The biological impact of these cytokines is modulated by the expression of their cellular receptors, which is influenced by genetic polymorphisms. However, the interplay between age, genetic variation, and cell-type-specific receptor expression remains incompletely characterized. This study aimed to determine the relative and absolute expression levels of IL-1 and TNF receptors on major immunocompetent cell populations in healthy donors of different age groups and to assess the influence of receptor gene polymorphisms on this expression. A cohort of 144 healthy donors was stratified into two age clusters using unsupervised clustering: a “young” group (18–31 years, n = 71) and an “older” group (32–59 years, n = 73). Membrane expression of TNFR1, TNFR2, IL-1R1, and IL-1R2 on T-lymphocytes, B-lymphocytes, and monocytes was analyzed by flow cytometry. The analysis included both the percentage of receptor-positive cells and the number of receptors per cell using absolute quantification with calibration beads. Genotyping for eight SNPs in the TNF1, TNFR2, IL1R1, and IL1R2 genes was performed via PCR-RFLP. The most pronounced age-related differences were observed in monocytes, in which the young cohort exhibited a significantly higher percentage of TNFR1- and TNFR2-positive monocytes, as well as a higher number of IL-1R1 receptors. In contrast, T-lymphocytes from the older cluster showed a higher percentage of TNFR2-positive cells. Genetic polymorphisms significantly modulated receptor expression in an age-dependent manner. For example, in the young cluster, polymorphisms primarily affected receptor levels on B-lymphocytes, whereas in the older cluster, the most significant associations were observed in monocytes. This study reveals significant, cell-specific alterations in the IL-1 and TNF receptor landscapes with age, with monocytes being particularly affected. The observed receptor downregulation in older adults is likely to reflect an active process of ligand-induced desensitization driven by chronic inflammation. Furthermore, genetic polymorphisms exert age-dependent effects on receptor expression, highlighting the dynamic interplay between genetics and immunosenescence. These findings provide a foundation for personalized strategies to mitigate inflammaging.

## 1. Introduction

Aging is a complex biological process characterized by the progressive deterioration of physiological functions. A central pillar of this process is a chronic, low-grade, systemic inflammatory state called “inflammaging” [1]. This phenomenon is a key driver of pathology, significantly increasing the risk of a spectrum of age-related diseases, including cardiovascular disorders, neurodegenerative conditions like Alzheimer’s, metabolic syndrome, and cancer [2,3,4]. Research shows that increases in inflammatory markers are non-specifically associated with aging [5]. Crucially, the biological impact of these cytokines is not solely dependent on their concentration but is profoundly shaped by the expression and function of their cellular receptors, which are, in turn, influenced by gene variants. A growing body of evidence now solidifies the link between polymorphisms in the genes encoding IL-1 and TNF receptors and the susceptibility to, and progression of, age-related decline [6,7].

The IL-1 family exerts some of the most potent inflammatory effects in the body, influencing everything from fever and leukocyte recruitment to tissue remodeling [8,9]. Its signaling is tightly regulated by a receptor duo—the signaling-competent IL-1 Receptor Type 1 (IL-1R1) and the non-signaling IL-1 Receptor Type 2 (IL-1R2)—the latter acting as a molecular “decoy” to sequester IL-1 and dampen its activity. The critical balance between these receptors determines the magnitude of IL-1 signaling. This balance is not identical in all individuals as it is modulated by genetics. Specific single-nucleotide polymorphisms (SNPs) in the IL1R1 and IL1R2 genes have been directly correlated with altered expression levels of their respective membrane-bound receptors [10]. This means that an individual’s genetic code can predispose them to a hyperactive or hypoactive IL-1 response. The clinical relevance of this is clear: certain polymorphisms in these receptor genes have been associated with an increased risk of age-related hearing impairment, providing a direct molecular link between genetic variation in the IL-1 pathway and a common age-related condition [11].

TNF-α similarly plays an important role in inflammation and cellular homeostasis. Its effects are channeled through two distinct receptors: TNF Receptor 1 (TNFR1), which is ubiquitous and often triggers pro-inflammatory and apoptotic pathways, and TNF Receptor 2 (TNFR2), which is more restricted in expression and is frequently associated with cell survival, proliferation, and immunoregulation [12,13]. The aging immune system exhibits dysregulated expression dynamics for these receptors, and research shows nonlinear, age-dependent shifts in the percentage of cells expressing TNFR1 and TNFR2 on immune cells, disrupting the delicate balance between destructive and protective TNF signaling and thereby fueling the inflammaging process [14,15]. Adding another layer of complexity, genetic polymorphisms in the TNFR1 and TNFR2 genes directly affect the expression levels of these receptors on immune cells [16,17,18]. This genetic variation contributes to the considerable heterogeneity observed in how different individuals experience inflammatory aging.

The downstream consequences of this dysregulated cytokine–receptor axis are profound and multifaceted. Meta-analyses confirm that elevated circulating levels of IL-1β and TNF-α are consistently associated with a higher prevalence of numerous age-related diseases, including cardiometabolic and endocrine disorders, neurodegenerative and cognitive conditions, cancer, and autoimmune diseases [19]. At the cellular level, this chronic inflammatory environment drives immunosenescence—the aging of the immune system. For example, naïve and central memory CD8+ T-cells from older adults show heightened susceptibility to TNF-α-induced apoptosis, a mechanism that depletes the repertoire of adaptive immune cells and compromises the ability to fight new infections and malignancies [20]. Beyond immune exhaustion, the pro-inflammatory milieu creates a fertile ground for cancer development. Notably, IL-1-driven inflammation has been shown to promote aging-associated oncogenesis in the lung, directly linking the molecular mechanisms of inflammaging to tumorigenesis [21].

With age, chronic, low-grade systemic inflammatory activity (inflammaging) leads to dysregulation and a persistently elevated background of IL-1 and TNF. This chronic stimulation becomes pathological and contributes significantly to the development of age-related diseases. Excess IL-1 and TNF is associated with neuroinflammation and cognitive decline, insulin resistance, sarcopenia, osteoporosis, and atherosclerosis. Thus, IL-1 and TNF exemplify the dichotomic nature of aging: molecules essential for survival and protection in youth, upon prolonged and dysregulated activation, transform into primary drivers of the pathological processes that underpin aging and age-related diseases [22].

An individual’s genetic profile for IL1R1, IL1R2, TNFR1, and TNFR2 sets a baseline for inflammatory responsiveness. With advancing age, this system becomes dysregulated, creating a self-perpetuating cycle of chronic inflammation that accelerates immunosenescence, increases cellular vulnerability, and fosters an environment conducive to age-related diseases and cancer. A detailed understanding of these genetic and molecular pathways is paramount for developing personalized, targeted anti-inflammatory interventions aimed at mitigating the burdens of inflammaging and promoting healthier longevity [6].

In conclusion, we hypothesize that the relationship between IL-1, TNF-α, and aging is deeply mechanistic, mediated through their specific receptor systems and modulated by genetic polymorphisms. Given the complex and poorly understood relationship between IL-1/TNF receptor expression, their polymorphisms, and age, the aim of this study is to determine the relative and absolute expression levels of these receptors on the main populations of immunocompetent cells in healthy donors of different age groups.

## 2. Results

### 2.1. Optimal Cluster Selection via AIC, BIC, and Silhouette Score Analysis

**Figure 1:**
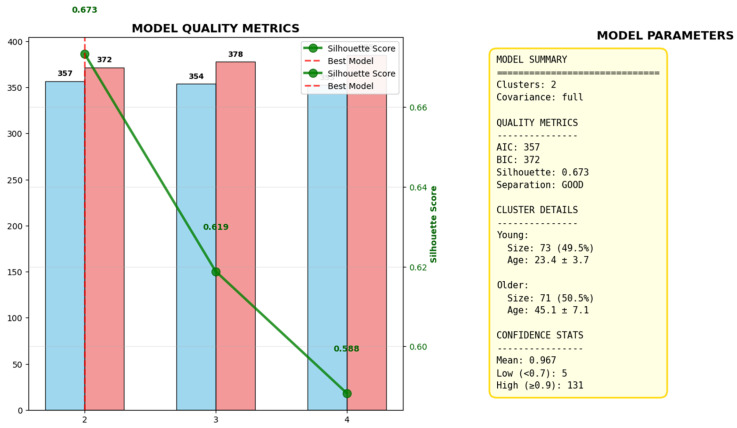
Evaluation of clustering metrics for determining the optimal number of age-based clusters. The figure presents the results of an unsupervised clustering analysis using Gaussian Mixture Models to identify the optimal number of demographic clusters within the study cohort. The evaluation is based on three standard metrics for assessing clustering quality: the Bayesian Information Criterion (BIC, blue bars), Akaike Information Criterion (AIC, pink bars), and the Silhouette Score (green line).

The Silhouette Score measures how similar an object is to its own cluster compared to other clusters, with higher values indicating better-defined clusters. The silhouette score is highest for the two-cluster (k = 2) solution (Figure 1).

The consistent convergence of all three metrics demonstrates that partitioning the study population into two distinct clusters provides the most robust and well-defined grouping for subsequent age-stratified analysis (Figure 1).

### 2.2. Demographic Characterization of Identified Clusters

The initial analysis included 150 samples, and following data preprocessing and excluding samples with incomplete records, the final analytical cohort consisted of 144 samples. Participants were included in the study if they met the following conditions:

No acute infectious or chronic inflammatory diseases at the time of enrollment and blood sampling.

No use of immunomodulatory, anti-inflammatory, or hormonal medications for a minimum of four weeks prior to the study.

Fulfillment of standard blood donor eligibility requirements, including stable hemoglobin levels and normotensive blood pressure.

Exclusion Criteria:

Participants were excluded from the study based on the following criteria:

A documented history of major chronic systemic diseases (e.g., cardiovascular, renal, hepatic, autoimmune, or oncological disorders).

A known history of transfusion-transmissible infections (e.g., HIV, hepatitis B virus, hepatitis C virus, or syphilis).

Administration of any vaccine within four weeks prior to blood collection.

Current pregnancy or lactation.

A history of major surgery or significant trauma within six months preceding the study.

Any other medical condition, ongoing treatment, or circumstance that, in the investigator’s judgment, could compromise the integrity of the study data or the participant’s safety.

Peripheral venous blood samples (9 mL) were collected from each participant at the blood collection center of City Clinical Hospital No. 1. All procedures were performed under strict aseptic conditions following the acquisition of written informed consent. Each sample tube was labeled with a unique donor identifier, sex, and age.

**Figure 2:**
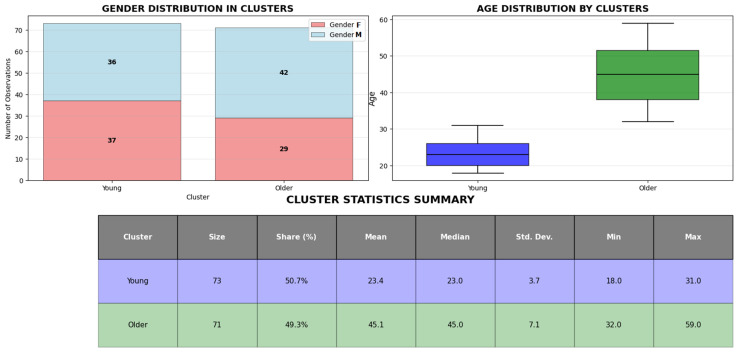
Demographic profile of the identified age-stratified clusters. The figure visually represents the distribution of individuals within these clusters, including the sample size (n) for each group. It also depicts the gender distribution (male/female) within each cluster, showing that both groups comprised a mix of male and female participants. This comprehensive demographic profile establishes the foundation for all subsequent comparative analyses of receptor expression and genetic associations between these two distinct age groups.

The unsupervised clustering analysis segregated the samples into two distinct demographic clusters: Cluster young (n = 71), had an age range of 18 to 31 years (mean = 23.4 years), and Cluster older (n = 73), spanned ages 32 to 59 years (mean = 45.1 years). A detailed characterization of the clusters, including the gender distribution, is provided in Figure 2.

### 2.3. Comparative Analysis of IL-1 and TNF Receptor Family Expression

The expression of IL-1 and TNF receptors was determined in peripheral blood mononuclear cells (PBMCs) isolated from venous blood samples of the healthy donors.

The detection method was flow cytometry using a panel of fluorescently labeled antibodies against the receptors (anti-TNF RI PE, anti-TNF RII PE, anti-IL-1 RI PE, anti-IL-1 RII PE) combined with antibodies for cell population identification (CD3 APC for T-cells, CD14 FITC for monocytes, CD19 PE-Cy7 for B-cells). Absolute receptor quantification was performed using BD QuantiBRITE PE calibration beads.

Significant inter-cluster differences were identified in the expression patterns of cytokine receptors from the IL-1 and TNF families.

#### 2.3.1. Analysis of TNF Receptor Family Expression

The frequency of TNFR1/2-positive blood cells was determined by flow cytometry analysis based on the percentage of receptor-positive cells within specific immune cell populations (T-lymphocytes, B-lymphocytes, and monocytes).

**Figure 3:**
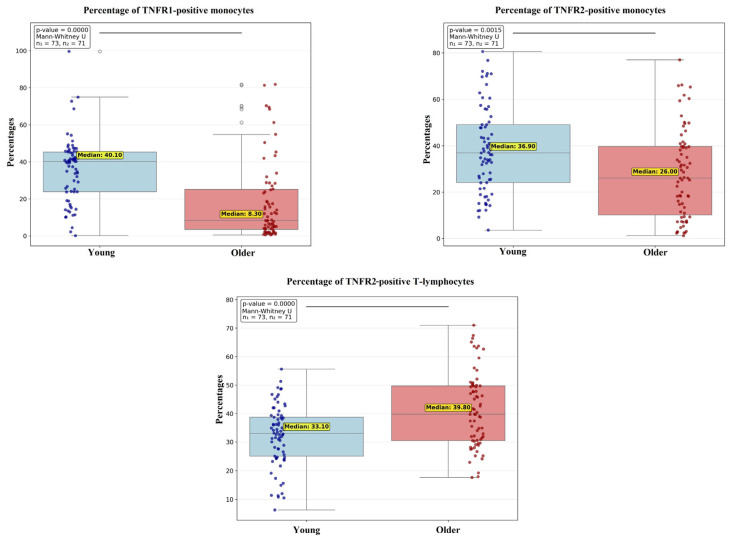
Comparison of the percentage of monocytes and T lymphocytes expressing TNFR1 and TNFR2 among peripheral blood mononuclear cells (PBMCs) between young and older clusters. Data are presented as median and interquartile range and outliers (white circles). Connecting lines between clusters indicate comparisons where *p* < 0.05.

Monocytes: The frequency of TNFR1-positive monocytes was significantly elevated in the young cohort compared to the older cohort (medians: 40.1% vs. 8.3%, *p* < 0.001; Figure 3). A similar disparity was observed for TNFR2, with a higher prevalence of TNFR2-positive monocytes in the young group (medians: 36.9% vs. 25.0%, *p* = 0.001; Figure 3).

T-Lymphocytes (Figure 3): In contrast, the analysis of the T-lymphocytes revealed an inverse pattern for TNFR2, in which the older cohort demonstrated a significantly greater percentage of TNFR2-positive T-cells (medians: 39.8% vs. 33.1%, *p* < 0.001; Figure 3).

No statistically significant inter-cluster differences were detected for the other leukocyte populations or soluble factors analyzed.

#### 2.3.2. Analysis of IL-1 Receptor Family Expression

**Figure 4:**
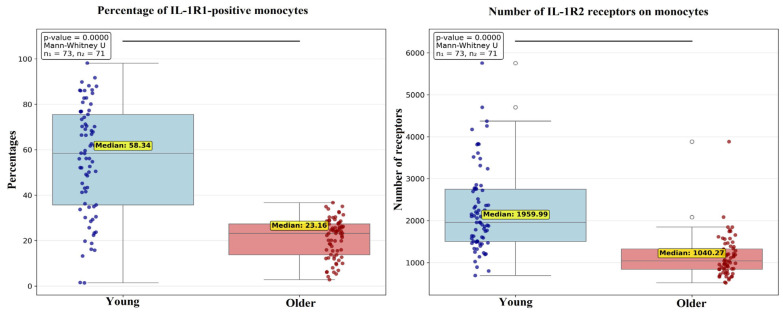
Comparison of the percentage of monocytes expressing IL-1R1 and numbers of IL-1R2 molecules among peripheral blood mononuclear cells (PBMCs) between young and older clusters. Data are presented as median and interquartile range and outliers (white circles). Connecting lines between clusters indicate comparisons where *p* < 0.05.

Significant age-associated differences in IL-1 receptor family expression were confined to monocytes, in which the young cluster exhibited a significantly higher percentage of IL-1R1-positive monocytes and a greater quantity of receptors per cell (Figure 4).

In summary, the age-stratified clusters demonstrate significant disparities in receptor expression on immunocompetent cells, affecting both pro-inflammatory cytokine receptor families (TNF and IL-1). Notably, monocytes displayed the greatest number of age-related differences.

We next performed a comparative analysis of the clusters after their stratification by receptor gene polymorphism, evaluating differences in the percentage of receptor-expressing cells, the number of receptors per cell, and soluble mediator levels.

### 2.4. Comparative Analysis of TNF Receptor Expression Across Age-Stratified Clusters Categorized by Receptor Gene Polymorphisms

#### 2.4.1. Analysis of TNF Receptor Gene Polymorphisms and Their Association with Receptor Expression in Human Immune Cells

Genotyping was performed using PCR–RFLP (polymerase chain reaction–restriction fragment length polymorphism) on genomic DNA isolated from peripheral blood mononuclear cells (PBMCs) of the study participants.

The rationale for selecting these specific single-nucleotide polymorphisms (SNPs) for investigation was grounded in their frequency and prior clinical associations.

Each SNP demonstrated a minor allele frequency (MAF) ≥ 1% in global populations, as catalogued in the NCBI dbSNP database. This criterion ensures their relevance for population-based genetic association studies and increases the likelihood of detecting significant phenotypic effects within a cohort.

The selection was further guided by existing literature indicating associations between these genetic variants and susceptibility to immune-mediated pathologies [11,23].

In this study, we investigated the association between Tumor Necrosis Factor Receptor 1 (TNFR1) gene polymorphisms (rs4149570 and rs149569) and TNF receptor expression patterns on immune cells across different age groups. For each polymorphism, carriers of different genotypes were compared based on the percentage of receptor-positive cells and the number of receptors per cell.

##### The Results for Polymorphism rs4149570 (Comparison of GG vs. GT Genotypes) (Figure 5)

In the older age cohort, a significantly higher percentage of TNFR2-positive T-lymphocytes was observed in individuals with the GG genotype compared to those with the GT genotype (46% and 39% *p* = 0.02). Similarly, the percentage of TNFR2-positive monocytes was significantly elevated in the GG genotype group versus the GT genotype group (33% and 16% *p* = 0.02).

In the young age cohort, differences were noted in the number of receptors.

The number of TNFR2 proteins on B-lymphocytes was significantly higher in individuals with the GT genotype compared to those with the GG genotype (1184 and 1021 *p* = 0.03).

##### The Results for Polymorphism rs149569 (Comparison of GG vs. GC Genotypes) (Figure 5)

In the young age cohort, a significant difference was found in the number of receptors. The number of TNFR1 proteins on monocytes was significantly higher in carriers of the GC genotype compared to those with the GG genotype (1008 and 820 *p* = 0.005).

##### The Results for Polymorphism rs 590368 (Comparison of CT vs. CC Genotypes) (Figure 6)

**Figure 6:**
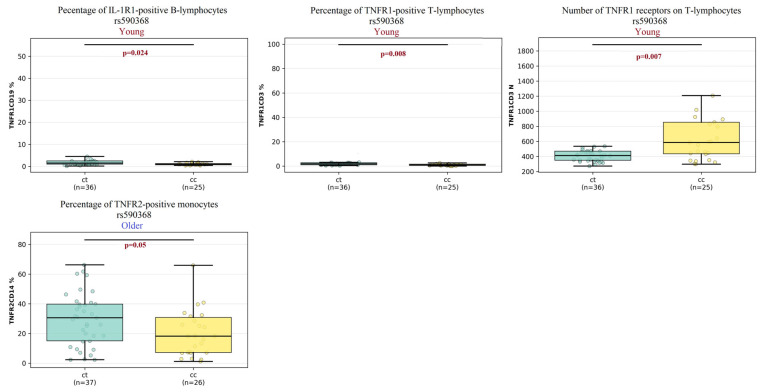
Comparison of the percentage of TNFRI and TNFR2-expressing cells and numbers of TNFR1 molecules among individuals with different genotype combinations of the rs590368 SNP between young and older clusters. Data are presented as median and interquartile range. Connecting lines between clusters indicate comparisons where *p* < 0.05.

Of the two TNFR2 polymorphisms investigated (rs652625 and rs590368), only rs590368 demonstrated significant associations with differential receptor expression patterns across the analyzed clusters (Figure 6).

In the elderly cluster, a significant difference was observed specifically in the percentage of TNFR2+ monocytes, in which individuals with the CT genotype exhibited a significantly higher percentage of TNFR2-expressing monocytes compared to those with the CC genotype (30% vs. 18%, *p* = 0.05).

Thus, in the young cluster, significant genotypic differences were identified for both the percentage of TNFR1+ T-lymphocytes and the number of receptors on these cells. The percentage of TNFR1+ T-lymphocytes was significantly higher in the CT genotype group compared to the CC group (1.9% vs. 0.9%, *p* = 0.008); conversely, the number of TNFR1 receptors per T-lymphocyte was significantly lower in the CT group compared to the CC group (413 vs. 586, *p* = 0.007). A significant association was also found for the percentage of TNFR1+ B-lymphocytes, which was higher in the CT genotype group compared to the CC group (1.5% vs. 1%, *p* = 0.02). Furthermore, within the young cluster, the number of TNFR2 receptors on T-lymphocytes also differed significantly: the TT genotype was associated with a higher number of TNFR2 receptors compared to the CT genotype.

#### 2.4.2. Analysis of IL-1 Receptors Gene Polymorphisms and Their Association with Expression of IL-1 Receptors in Human Immune Cells

##### IL-1 Gene Polymorphisms

We also investigated the association between IL-1 gene polymorphisms and IL-1 receptor expression on immunocompetent cells in two clusters. For the rs3917225 and rs2234650 polymorphisms, carriers of different genotypes were compared based on the percentage of receptor-positive cells and the number of receptors per cell.

**Figure 7:**
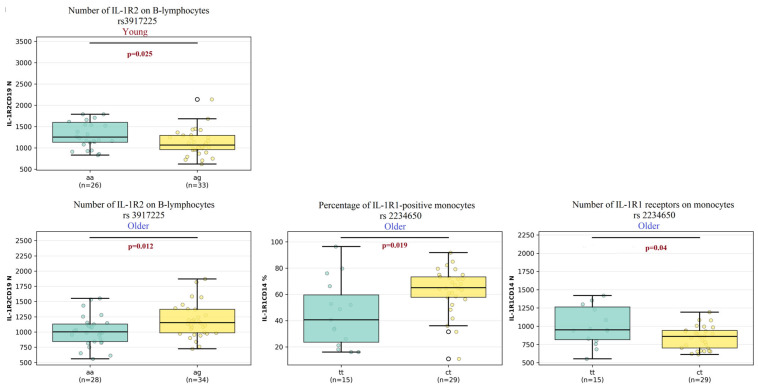
Comparison of the percentage of IL-1R1-expressing cells and numbers of IL-1R1 and IL-1R2 molecules among individuals with different genotype combinations of the rs3917225 and rs2234650 SNPs between young and older clusters. Data are presented as median and interquartile range. Connecting lines between clusters indicate comparisons where *p* < 0.05.

Significant associations for the IL-1R1 polymorphism rs3917225 were observed in both age clusters (Figure 7), specifically affecting IL-1 receptor expression on B-lymphocytes.

In the young cluster, the number of IL-1R2 receptors on B-lymphocytes was significantly higher in individuals with the AA genotype compared to those with the AG genotype (1253 vs. 1065, *p* = 0.02).

In the older cluster, a contrasting pattern was found for the number of IL-1R1 proteins on B-lymphocytes. Here, the AG genotype was associated with a significantly higher receptor level compared to the AA genotype (153 vs. 1003, *p* = 0.01).

The analysis of the rs2234650 polymorphism revealed significant differences exclusively within the older cluster, primarily affecting monocyte populations.

Both the frequency of IL-1R1-positive monocytes and the number of receptors per cell were significantly influenced by the genotype.

The percentage of IL-1R1+ monocytes was significantly higher in the TT genotype group compared to the CT genotype group (65% vs. 40%, *p* = 0.01).

Furthermore, monocytes from the TT genotype group exhibited a significantly greater number of IL-1R1 receptors than those from the CT genotype group (950 vs. 861, *p* = 0.04).

##### IL-1R2 Polymorphisms

**Figure 8:**
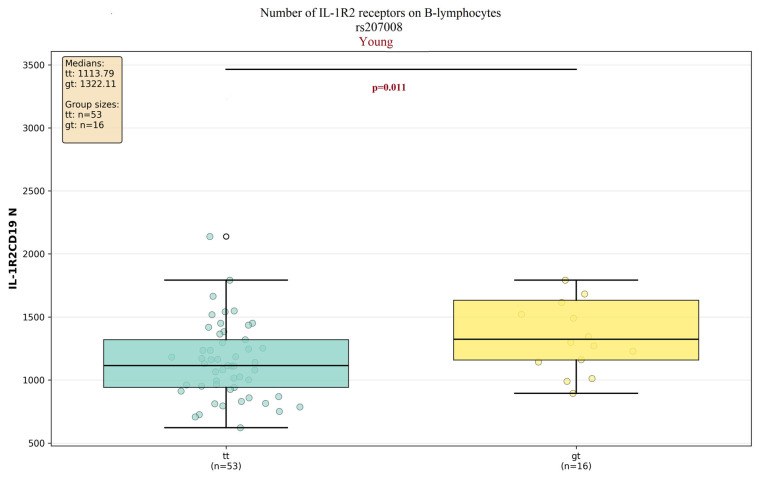
Comparison of the numbers of IL-1R2 molecules among individuals with different genotype combinations of the rs2071008 SNP between young cluster. Data are presented as median and interquartile range and outliers (white circles). Connecting lines between clusters indicate comparisons where *p* < 0.05.

Of the two IL-1R2 polymorphisms investigated (rs4141134 and rs2071008), significant associations were identified only for rs2071008; this association was specific to the young cluster and pertained exclusively to the number of IL-1R2 proteins on B-lymphocytes (Figure 8).

Individuals with the GT genotype exhibited a significantly higher receptor expression compared to those with the TT genotype (1322 vs. 1113, *p* = 0.01).

## 3. Discussion

This study used an unsupervised clustering approach to stratify a cohort of 144 individuals into two distinct age groups: a young cluster (18–31 years, mean 23.4) and an older cluster (32–59 years, mean 45.1). Our central finding is that these age-defined clusters exhibit significant and cell-specific differences in the expression of receptors belonging to the TNF and IL-1 pro-inflammatory cytokine families. The most pronounced age-related differences were consistently observed in the monocyte population, which reveals a complex and compartmentalized reshaping of the immune landscape with age [24].

The most notable observation from our data is the predominant effect of age on monocyte receptor phenotypes. We found that young individuals displayed a significantly higher percentage of monocytes expressing both TNFR1 and TNFR2, as well as a higher overall level of TNFR1 expression. This suggests a heightened readiness of the early adult immune system to respond to TNF-mediated signaling. TNF is a crucial regulator of inflammatory and anti-apoptotic pathways, and its effects are critically dependent on the balance between its two receptors [25]. TNFR1 often mediates pro-inflammatory and apoptotic signals, while TNFR2 is frequently associated with tissue repair and immunoregulatory functions.

Our observation that TNFR1 is elevated in youth appears to contrast with some studies reporting increased inflammation, or “inflammaging,” in older adults [26]; however, this discrepancy may be explained by a fundamental shift in receptor homeostasis. A compelling mechanism for the decreased TNFR surface expression in the older cohort is the age-associated dysregulation of receptor internalization and turnover. In robust, young immune systems, ligand-induced receptor internalization is a tightly regulated process that serves to attenuate signaling and allow for receptor recycling or degradation. With advancing age, however, chronic, low-grade inflammation—characterized by persistently elevated levels of circulating TNF-α—can lead to a state of perpetual receptor activation and subsequent internalization. Studies have shown that continuous exposure to TNF-α promotes the sustained internalization of TNFR1 into endosomal compartments, where it is targeted for lysosomal degradation rather than recycled to the plasma membrane [27,28,29]. This phenomenon, driven by the chronic inflammatory milieu of aging, results in a net reduction of surface-available TNFR1, as observed in our older cohort. Therefore, the observed decrease is not merely a passive loss but likely an active, maladaptive process of chronic stimulation, rendering monocytes less responsive to acute TNF challenges—a state of immune tolerance.

In this study, T-lymphocytes exhibited the inverse phenomenon, with a significantly greater percentage of TNFR2+ cells in the older cluster. This cell-type-specific regulation underscores the complexity of immunosenescence. On monocytes, the co-downregulation of TNFR1 and TNFR2 with age supports the hypothesis of generalized TNF receptor desensitization, potentially driven by the same mechanisms of chronic internalization. On the other hand, the upregulation of TNFR2 on T-cells could represent a distinct, compensatory mechanism or a marker of immunological aging. TNFR2 is a key costimulatory molecule for regulatory T-cells (Tregs) and is involved in the expansion and survival of this immunosuppressive population. Age-associated expansion of TNFR2+ T-cells has been linked to the accumulation of senescent T-cell phenotypes and a progressive decline in adaptive immune efficacy [14]. Thus, the increased quantity of TNFR2 on T-cells in the older cluster may not signify enhanced immunity but rather a shift towards a more immunoregulatory T-cell pool [30].

The observed differences were not confined to the TNF superfamily. A parallel reduction in both the percentage of IL-1R1+ monocytes and the level of its expression was found in the older cohort. The IL-1 family is another cornerstone of immunity, with IL-1β being a potent pyrogen and driver of inflammation. The coordinated downregulation of receptors for two major pro-inflammatory pathways (TNF and IL-1) strongly points toward a fundamental reprogramming of monocyte function [31]. The mechanism for IL-1R reduction may similarly involve ligand-induced internalization and degradation, as IL-1R signaling is also subject to tight regulation via endocytic processes [32]. This global dampening of primary inflammatory receptors could be a protective mechanism against chronic inflammation but comes at the cost of reduced vigilance against new infections.

Our results contribute a nuanced perspective to the concepts of “inflammaging” and immunosenescence. The classical view of inflammaging emphasizes a progressive increase in circulating pro-inflammatory cytokines [33]; however, our data highlight that the receptor landscape—the cellular “sensors” for these cytokines—undergoes significant alteration. We propose a model where systemic inflammaging (elevated circulating cytokines) directly drives the internalization and downregulation of receptors on immune cells like monocytes. This creates a paradoxical state of both chronic inflammation, as measured by plasma cytokines, and cellular immune deficiency, as measured by receptor availability and, likely, functional responsiveness.

The lack of significant differences in the other cell populations and soluble factors analyzed in our study further underscores these conclusions. It suggests that the monocyte compartment is particularly vulnerable to this age-related reprogramming, acting as a primary conductor of the immunological changes that characterize the transition from young adulthood to middle age.

Aging is associated with the accumulation of epigenetic modifications, such as DNA hypermethylation and altered histone acetylation, which can silence gene promoters. The promoters of TNFR1 and TNFR2 may become less accessible to transcription factors over time, leading to reduced mRNA production. This is supported by broader studies on immunosenescence showing that age-related epigenetic drift significantly impacts the expression of critical immune response genes [34,35,36]. Furthermore, chronic low-grade inflammation (inflammaging) can lead to a desensitization of key transcription factors like NF-κB, paradoxically resulting in the downregulation of certain target genes, including receptors, as part of a feedback mechanism to limit excessive signaling [37,38].

At the post-transcriptional level, regulatory mechanisms further modulate receptor levels. The stability and translation of TNFR mRNA can be influenced by age-associated microRNAs (miRNAs). For instance, miR-146a, a key inflammation-responsive miRNA that is often elevated with age, has been shown to target and repress the expression of various components of the immune signaling pathways, potentially including the mRNAs for TNF receptors, thereby contributing to their decreased synthesis [39]. This represents a layer of regulation where the inflammatory milieu of aging directly fine-tunes receptor availability.

The stability of the receptors themselves is also a critical factor. The process of proteolytic shedding, where membrane-bound receptors are cleaved by metalloproteinases like ADAM17 to release soluble forms (sTNFR), is a key regulatory node [40]. With advancing age, increased proteolytic activity or expression of these sheddases could lead to accelerated cleavage of TNFR1 and TNFR2 from the monocyte surface, effectively reducing their membrane-bound levels while increasing their soluble counterparts in the circulation [41,42].

Finally, cell-intrinsic changes and shifts in monocyte subsets contribute to the observed phenotype. Aging drives remodeling of the hematopoietic system, leading to alterations in the output and phenotype of immune cells. There is a documented shift in monocyte subsets with age, often towards a more inflammatory classical phenotype [43]; however, these cells may exist in a state of chronic pre-activation or “exhaustion,” which can be associated with downregulation of certain surface receptors as a form of desensitization. Therefore, the overall reduction in TNFR expression across the monocyte pool may reflect both a change in the transcriptional program of individual cells and an alteration in the proportional representation of different subsets.

Our previous research has shown that receptor gene polymorphisms are associated with both the percentage of cells expressing these receptors and the number of receptors per cell [44]. Furthermore, studies have indicated that receptor expression on cells correlates with age and differs across TNF receptor gene polymorphisms [14]. In our current study, we demonstrate that the two age clusters, when further stratified by TNF and IL-1 receptor gene polymorphisms, show differences in receptor expression levels on various immune cells. Table 1 presents data on the frequency of specific polymorphisms and their associated changes in receptor expression.

For the young cluster, significant differences were observed in both the quantity and percentage of B-lymphocytes expressing TNF and IL-1 receptors; in contrast, the older cluster exhibited more pronounced differences in monocytes. These disparities may be attributed, first, to differences in subpopulation composition. It has been shown that the absolute count of B-cells is higher in young individuals compared to older age groups, while the percentage of naïve B-cells is significantly reduced in older groups, with an increased percentage of atypical memory B-cells [45]. Research has also demonstrated that the expression of both types of TNF receptors and IL-1 receptors varies across B-lymphocyte subpopulations [46,47]. The differences we obtained show the influence of polymorphisms in the genes for both cytokine receptors on receptor expression in B-lymphocytes, suggesting an important regulatory role for these cells during youth. This is manifested, for example, in a better response to vaccination [48].

Our data indicate that cytokine receptor gene polymorphisms are associated with differences in receptor expression on monocytes in the older cohort. Monocytes also undergo age-related changes in their subpopulation composition; studies show that as adults age, the frequency and absolute count of intermediate and non-classical monocyte subsets increase [31]. Furthermore, it has been shown that different monocyte subpopulations express different levels of TNF receptors, which influences their differentiation and functions [49,50]. Monocytes isolated from older individuals have been found to produce larger amounts of pro-inflammatory cytokines [51].

While our study provides valuable insights, several limitations should be acknowledged. First, the cohort size of 144 individuals, while sufficient for the initial clustering, may limit the statistical power for more detailed subgroup analyses, especially when stratifying by both age cluster and multiple genetic polymorphisms. Second, our analysis focused on surface receptor expression and genetic variation but did not include direct functional assays to measure downstream signaling activity (e.g., NF-κB activation) or cellular responses to ligand stimulation. Consequently, the functional consequences of the observed receptor changes are inferred from the literature rather than directly demonstrated. Third, the age range of our “older” cluster (32–59 years) captures early to middle adulthood and may not fully represent the more pronounced immunological alterations characteristic of advanced age (e.g., 65+ years). Finally, while we discuss potential mechanisms such as epigenetic regulation and receptor shedding, these were not directly measured in our study, and their specific contributions remain to be experimentally validated.

## 4. Materials and Methods

### 4.1. Study Cohort and Sample Collection

The study cohort comprised 150 healthy, unrelated blood donors of Russian ethnicity from Novosibirsk, Western Siberia (83 males, 67 females; mean age 34 ± 1 years, range 19–55 years). Venous blood samples were collected after overnight fasting using VACUETTE tubes containing K3-EDTA anticoagulant (Greiner Bio-One, Vacuette GmbH, Frankhauser, Germany) for cellular studies and serum clot activator tubes for serum isolation. All participants underwent comprehensive health screening according to Russian Federation blood donor standards, with exclusion criteria including acute or chronic inflammatory diseases, recent medication use, abnormal vital signs, and pregnancy.

### 4.2. PBMC Isolation

Peripheral blood mononuclear cells were isolated using density gradient centrifugation with Ficoll–Urografin (Pharmacia Fine Chemicals, Stockholm, Sweden, and Schering AG, Berlin, Germany, respectively) solution (ρ = 1.077 g/cm^3^).

### 4.3. Flow Cytometric Analysis of Membrane Receptors

A flow cytometry panel was established for simultaneous quantification of membrane-bound receptors and immune cell phenotyping (Table S1). The antibody panel included anti-TNF RI PE, anti-TNF RII PE, anti-IL-1 RI PE, and anti-IL-1 RII PE (R&D Systems, Minneapolis, MN, USA), Figure S3E,F and Figure S4A,B combined with anti-CD3 APC, anti-CD14 FITC, and anti-CD19 PE-Cy7 (eBioscience, San Diego, CA, USA) for population identification (Figure S3A–D). Critical methodological optimizations included Fc receptor blocking with human IgG (NPO Microgen, Moskow, Russia) and antibody titration to determine saturating concentrations. Absolute receptor quantification was achieved using BD QuantiBRITE PE calibration beads (BD Biosciences, Franklin Lakes, NJ, USA), with weekly instrument calibration using Cytometer Setup and Tracking beads (BD Biosciences, Franklin Lakes, NJ, USA, Figures S1 and S2). Samples were analyzed on a BD FACSAria flow cytometer (BD Biosciences, Franklin Lakes, NJ, USA, Table S2), collecting ≥ 10,000 events per sample, with data analysis focusing on lymphocyte and monocyte populations gated by forward/side scatter characteristics [52]. See Supplementary Materials.

### 4.4. Genetic Analysis

DNA Extraction and Quality Control: Genomic DNA was isolated from PBMCs using optimized phenol–chloroform extraction, with DNA quality verified by spectrophotometry and gel electrophoresis.

SNP Selection and Genotyping: Eight functionally relevant SNPs in promoter regions of TNF and IL-1 receptor genes were selected based on minor allele frequency (>10%) and potential regulatory effects. The genotyping strategy employed PCR-RFLP analysis with the following primer sets and conditions:

- TNFRI rs4149570 (−609G/T): Forward 5′-CGGACGCTTATCTATATCTC-3′, Reverse 5′-TTGTAGTCCAGTCACAAGCA-3′ (Bst4C I digestion).
- TNFRI rs4149569 (−1207C/G): Forward 5′-TTGGGAGATGTCTGCATCAA-3′, Reverse 5′-TTCTTCGTTTGCTTGTTTTTCA-3′ (BstC8 I digestion).
- TNFRII rs652625 (−709A/T): Forward 5′-GAGTGCTGAGTGAGAAACTG-3′, Reverse 5′-AGCTTGAATTCGTTCCCAGG-3′ (DseD I digestion).
- TNFRII rs590368 (−3609C/T): Forward 5′-ATGCTTTTGTCCATGCAGGT-3′, Reverse 5′-GCTGTACCCCGTATTAGCTG-3′ (Msp I digestion).
- IL1RI rs3917225 (−1100A/G): Forward 5′-TCTGGGGCATACTCACAGGGGT-3′, Reverse 5′-AGCTGGGTTGTGGTAGCCTTACTG-3′ (AsuHP I digestion).
- IL1RI rs2234650 (−12075C/T): Forward 5′-TTGGAGGATGGCCCATGAAGACC-3′, Reverse 5′-CTGTTACGCGCCCGGATGAAAAA-3′ (Pst I digestion).
- IL1RII rs4141134 (−1780C/T): Forward 5′-CCATGCCATCTGCTCTTGGCCAT-3′, Reverse 5′-GACCAGACTTTGGAAAGGCCTCC-3′ (Msp I digestion).
- IL1RII rs2071008 (+6974G/T): Forward 5′-CTTACATGGCTGGTGCCTTT-3′, Reverse 5′-TATCTCCCATCCCACATGGT-3′ (Dra III digestion).

PCR was carried out in a PTC-200 DNA Engine thermal cycler (MJ Research Inc., Watertown, NY, USA) with 20 µL reactions containing Taq DNA polymerase (SibEnzyme, Novosibirsk, Russia), 0.5 µM primers, 0.25 mM dNTPs, 50–200 ng genomic DNA, and buffer. The cycling conditions comprised an initial denaturation at 95 °C for 3 min; 30–35 cycles of 94 °C for 20 s, 58–64 °C for 15 s, and 72 °C for 20 s; and a final extension at 72 °C for 2 min. Amplicons were digested with appropriate restriction endonucleases (SibEnzyme, Novosibirsk, Russia) at 37–65 °C for 18 h. The fragments were separated via capillary electrophoresis (QIAxcel System, Qiagen, Hilden, Germany) or 2% agarose gel electrophoresis (140–150 V, 20–25 min) and visualized under UV light. Fragment sizing was performed with ImageMaster VDS software (Pharmacia Biotech, Uppsala, Sweden).

### 4.5. Statistical Analysis

Data were collected and analyzed using MS Excel 365 and JASP v. 0.95.4 (University of Amsterdam, Amsterdam, The Netherlands, 2025), and then were visualized using Python (version 3.14) with the following libraries: Matplotlib (version 3.10.7) for plotting, Pandas (version 2.3.3) for data handling, and NumPy (version 2.3.4) for numerical computations. We initially used Gaussian Mixture Models for age clustering; comparisons of different numbers of clusters are presented in Figure 1. Next, we compared expression and co-expression indices of receptors and soluble factor levels between clusters using the Kruskal–Wallis test for multiple group comparisons and the Mann–Whitney U test for pairwise comparisons. Multiple testing corrections were applied using the Bonferroni method, with a statistical significance threshold set at *p* < 0.05. Genetic analyses included Hardy–Weinberg equilibrium testing using the χ^2^ test and genotype–phenotype association studies.

## 5. Conclusions

This study demonstrates the complex and multifaceted influence of age and genetic variation on the expression of receptors for key pro-inflammatory cytokines, IL-1 and TNF, in the human immune system. The central finding is the identification of significant, cell-specific disparities in the expression of TNF (TNFR1 and TNFR2) and IL-1 (IL-1R1) receptors between two distinct age clusters (“young,” 18–31 years, and “older,” 32–59 years). The most pronounced age-related differences were consistently observed in the monocyte compartment. The young cohort exhibited a significantly higher percentage of monocytes expressing TNFR1 and TNFR2, as well as a greater number of IL-1R1 and TNFR1 receptors on these cells. This suggests a heightened baseline readiness for cytokine signaling in early adulthood. In contrast, the observed downregulation of these receptors in monocytes from the older group likely reflects an active, maladaptive process driven by chronic, low-grade inflammation (“inflammaging”), potentially involving ligand-induced internalization, degradation, and epigenetic silencing, leading to a state of cellular desensitization. Conversely, T-lymphocytes exhibited an inverse pattern for TNFR2, with a higher percentage of positive cells in the older cluster. This cell-type-specific regulation underscores the complexity of immunosenescence, possibly indicating a shift towards a more immunoregulatory T-cell pool. Furthermore, we established that genetic polymorphisms in the TNFR1, TNFR2, IL1R1, and IL1R2 genes significantly modulate receptor expression levels, and crucially, these genetic effects were often age-dependent. In the young cohort, polymorphisms primarily influenced receptor expression on B-lymphocytes, whereas in the older cohort, the most significant associations were observed in monocytes. This highlights that genetic control of the cytokine receptor landscape is not static but evolves with the aging immune system.

In summary, our results support a model in which systemic inflammation, characterized by elevated circulating cytokine levels, triggers compensatory downregulation of receptors on immune cells such as monocytes. This creates a paradoxical state of chronic inflammation coupled with cellular immunodeficiency. Individual genetic predisposition to these receptors sets a baseline inflammatory response that deteriorates with age, contributing to the heterogeneity of immunosenescence. A detailed understanding of these age- and genotype-dependent pathways is crucial for the development of personalized, targeted anti-inflammatory interventions aimed at reducing the burden of inflammation and promoting healthier longevity.

## Figures and Tables

**Figure 5:**
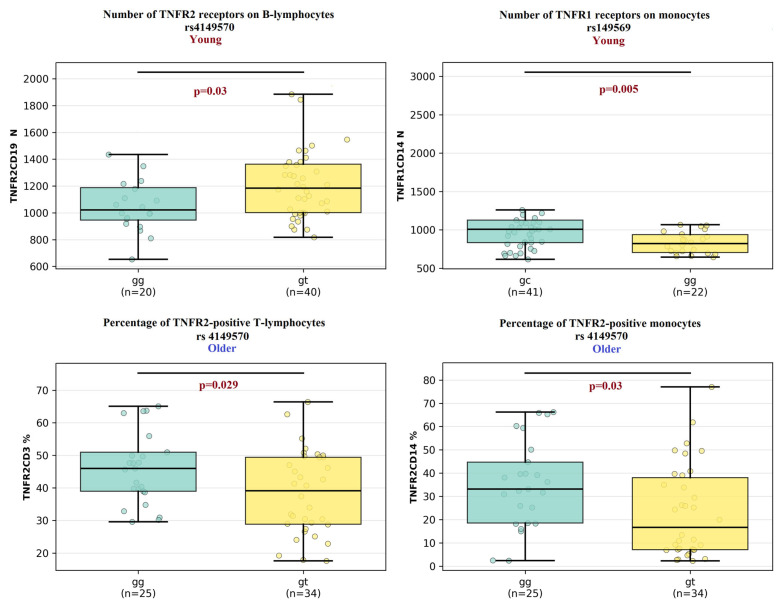
Comparison of the percentage of TNFRI and TNFR2-expressing cells among individuals with different genotype combinations of the rs4149570 and rs149569 SNPs between young and older clusters. Data are presented as median and interquartile range. Connecting lines between clusters indicate comparisons where *p* < 0.05.

**Table 1:** Summary of significant associations between cytokine receptor gene polymorphisms and cell-type-specific receptor expression across age groups. The symbols ↑ and ↓ indicate a statistically significant increase or decrease, respectively, in the receptor expression parameter (percentage of positive cells or receptor count per cell) for the first-listed genotype compared to the second in the “Genotype Comparison” column.

Polymorphism	Genotype Frequency, % (n)	Genotype Comparison	Age Group	Cell Type	Observed Effect
rs4149570	GG: 31.3% (45) GT: 51.4% (74)	GG vs. GT	Older	T-lymphocytes, monocytes	↑ % TNFR2+ cells
		GG vs. GT	Young	B-lymphocytes	↑ TNFR2 count per cell
rs4149569	GG: 36.1% (52) GC: 46.6% (67)	GG vs. GC	Young	Monocytes	↑ TNFR1 count per cell
rs590368	CC: 36.8% (53) CT: 48.6% (70)	CT vs. CC	Older	Monocytes	↑ % TNFR2+ cells
		CT vs. CC	Young	T- and B-lymphocytes	↑ % TNFR1+ cells, ↓ TNFR1/cell, ↑ TNFR2/cell
rs3917225	AA: 38.8% (56) AG: 45.9% (66)	AA vs. AG	Young	B-lymphocytes	↑ IL-1R2 count per cell
		AA vs. AG	Older	B-lymphocytes	↑ IL-1R1 count per cell
rs2234650	TT: 20.8% (26) CT: 57.6% (72)	TT vs. CT	Older	Monocytes	↑ % IL-1R1+ cells and ↑ receptor count per cell
rs2071008	TT: 79.1% (114) TG: 19.4% (28)	GT vs. TT	Young	B-lymphocytes	↑ IL-1R2 count per cell

## Data Availability

The original contributions presented in this study are included in the article/Supplementary Materials. Further inquiries can be directed to the corresponding authors.

## Supplementary Materials

The following supporting information can be downloaded at: https://www.mdpi.com/article/10.3390/ijms27020807/s1.

## Abbreviations

The following abbreviations are used in this manuscript:

IL-1	Interleukin-1
TNF-α	Tumor Necrosis Factor-alpha
IL-1R1	IL-1 Receptor Type 1
IL-1R2	IL-1 Receptor Type 2
TNFR1	TNF Receptor 1
TNFR2	TNF Receptor 2
SNP	Single-Nucleotide Polymorphism
PCR-RFLP	Polymerase Chain Reaction–Restriction Fragment Length Polymorphism
PBMC	Peripheral Blood Mononuclear Cell
EDTA	Ethylenediaminetetraacetic acid
PE	Phycoerythrin
APC	Allophycocyanin
PE-Cy7	Phycoerythrin-Cyanine 7
NF-κB	Nuclear Factor Kappa-Light-Chain-Enhancer of Activated B cells
miRNA	microRNA
ADAM17	A Disintegrin And Metalloproteinase 17
sTNFR	Soluble TNF Receptor
Tregs	Regulatory T cells
DNA	Deoxyribonucleic acid
BIC	Bayesian Information Criterion
AIC	Akaike Information Criterion
