# Supplementary material for: Age- and Genotype-Associated Specific Expression of IL-1 and TNF Receptors on Immunocompetent Cells
Source: Int J Mol Sci. 2026 Jan 13;27(2):807. doi: 10.3390/ijms27020807 (PMC12841339; doi:10.3390/ijms27020807)
Supplement: Supplementary file 1 [file ijms-27-00807-s001.zip › ijms-4033894-supplementary.pdf]

Minimum Information about a Flow Cytometry Experiment (MIFlowCyt)

Experiment Annotation

Funding: This research was funded by the Russian Science Foundation through award #25-25-00120 (https://rscf.ru/project/25-25-00120/).

Introduction

This document represents an example description of a flow cytometry experiment that is compliant with the Minimum Information about a Flow Cytometry Experiment (MIFlowCyt).

1. Experiment Overview

1.1. Purpose

The aim of this study is to determine the relative and absolute expression levels of these re-ceptors on the main populations of immunocompetent cells in healthy donors of different age groups.

1.2. Keywords

Blood, T- lymphocyte, monocyte, B-lymphocyte, receptors

1.3. Experiment Variables

We used aliquots of whole blood with a leukocyte content of 500 thousand/ml for staining for each sample.

Quality Control Measures

Control was carried out on unpainted samples.

2. Flow Sample/Specimen Details

2.1. Sample/Specimen Material Description

2.1.1. Biological Samples

Peripheral blood collection from conditionally healthy individuals was performed in the morning after an overnight fast from the cubital vein under strict sterile conditions. Nine milliliters of peripheral blood were collected into a VACUETTE vacuum tube containing K3-EDTA as an anticoagulant ("Greiner Bio-One," Austria). Peripheral blood mononuclear cells (PBMCs) from conditionally healthy individuals were isolated using the standard density gradient centrifugation method with Ficoll ("Pharmacia Fine Chemicals," Sweden) – Urografin ("Schering AG," Germany) (density 1.077 g/cm³) [Böyum 1968]. The blood was diluted 1:1 with RPMI-1640 culture medium (LLC "BioloT," Saint Petersburg, Russia). Then, 5 mL of the diluted blood was layered onto 3 mL of the Ficoll-Urografin solution and centrifuged at 3000 rpm for 20 minutes. After centrifugation, the cells from the interphase ring were collected and transferred into RPMI-1640 medium to remove any residual Ficoll-Urografin. The cells were centrifuged at 1000 rpm for 10 minutes, and the supernatant was removed. This washing step was repeated twice. The pellet was resuspended in complete RPMI-1640 culture medium containing 10% FCS ("HyClone," USA), 2 mM L-glutamine (LLC "BioloT," Saint Petersburg, Russia), 5×10^{-4} M 2-mercaptoethanol ("Sigma-Aldrich," USA), 10 mM HEPES buffer ("Sigma-Aldrich," USA), 80 µg/mL gentamicin ("KRKA," Slovenia), and 100 µg/mL benzylpenicillin (JSC "Biosintez," Penza, Russia).

2.2. Sample Treatment Description

The percentage of cells expressing membrane-bound receptors for TNF-alpha and IL-1 type I and II was determined by flow cytometry. The following phycoerythrin (PE)-conjugated antibodies from "R&D Systems" (USA) were used for the method: anti-human TNF RI PE, anti-human TNF RII PE, anti-human IL-1 RI PE, and anti-human IL-1 RII PE. For immunophenotyping subpopulations of peripheral blood mononuclear cells, antibodies from "eBioscience" (USA) were used: anti-CD3 APC, anti-CD14 FITC, and anti-CD19 PE-Cy7.

A calibration curve was constructed, and the fluorescence intensity values of cells expressing the corresponding marker were converted into absolute receptor numbers using the BD QuantiBRITE PE kit ("BD Biosciences", USA). This kit contains 4 fractions of lyophilized beads, each coated with a different known level of phycoerythrin. The bead tube was reconstituted in 500 µl of 1× PBS (137 mM NaCl, 2.68 mM KCl, 10 mM $Na_2HPO_4 \cdot 12H_2O$, 1.47 mM KH_2PO_4, 0.53 mM EDTA, and 0.1% NaN_3), mixed, and analyzed on a BD FACSAria flow cytometer ("BD Biosciences", USA). On the FSC-A/SSC-A dot plot, the bead population was gated, and 10,000 events were recorded. Subsequently, markers were set on the PE fluorescence histogram based on the four peaks of the calibration particles (Low, Med Low, Med High, High). The analysis of the BD QuantiBRITE PE calibration particles is shown in Figure S1.

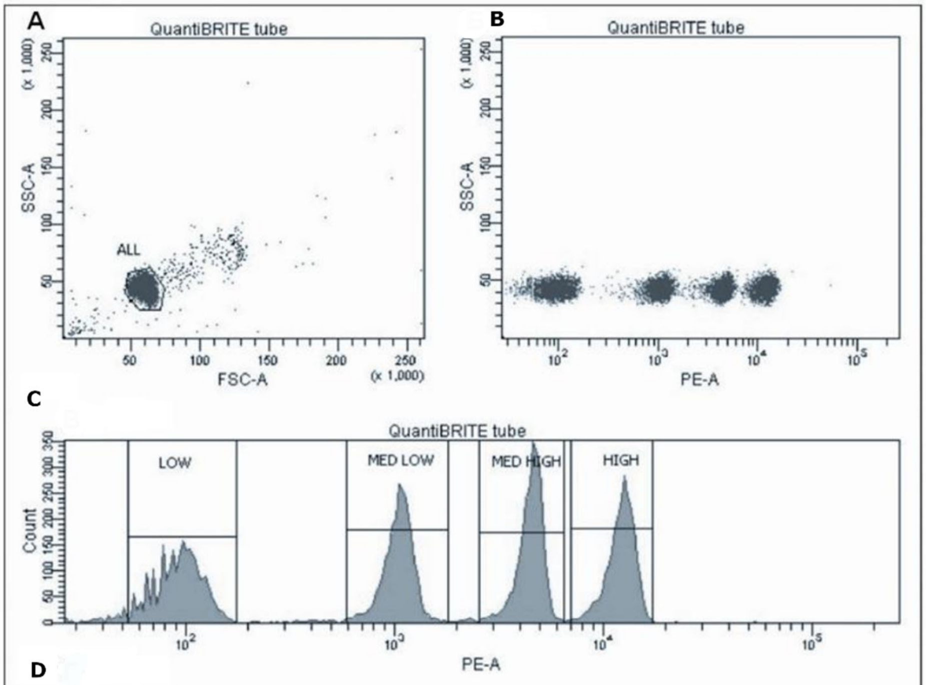

D

Population	#Events	PE-A Min	PE-A Max	PE-A Geo Mean
LOW	2,395	54	177	95
MED LOW	2,468	588	1,738	1,055
MED HIGH	2,596	2,552	6,446	4,536
HIGH	2,428	7,150	17,581	12,335

Figure S1. Analysis of QuantiBRITE PE calibration particles ("BD Biosciences", USA). (A) Gating of calibration particles on the FSC-A/SSC-A dot plot. **(B)** Calibration particles on the PE/SSC-A dot plot. **(C)** PE fluorescence histogram. Markers are set based on the four peaks of the calibration particles (Low, Med Low, Med High, High). **(D)** Statistics.

Based on the bead analysis results, a graph plotting the logarithmic values of the number of phycoerythrin molecules against fluorescence intensity was constructed, establishing a mathematical linear logarithmic relationship (Figure S2).

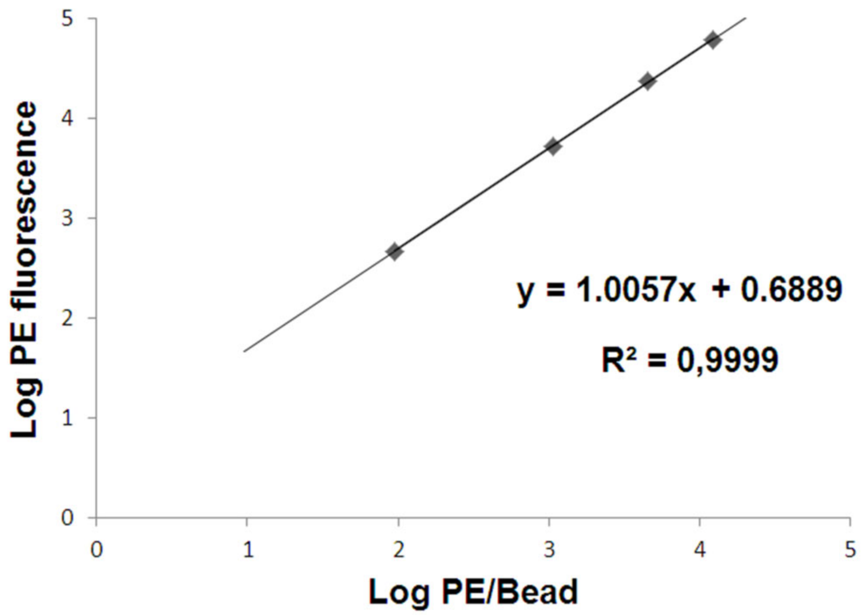

Figure S2. BD QuantiBRITE PE calibration curve. The dependence of logarithmic values of the number of PE molecules on fluorescence intensity.

Using the derived formula and the fluorescence intensity values of each subpopulation, the absolute numbers of receptors per cell were calculated.

Construction of the calibration curve.

PE fluorescence geometric means	Log PE fluorescence	PE molecules/ bead*	Log PE molecules/bead
95	1.977723605	474	2.675778342
1058	3.024485668	5359	3.729083757
4575	3.660391098	23843	4.377360899
12411	4.093806776	62336	4.794738931

*Lot-specific values for the number of PE molecules per bead.

To achieve optimal stability and experimental reproducibility, we established an optimal sample preparation protocol for determining the expression level of membrane-bound TNF-alpha and IL-1 receptors. Before the experiment, the saturating antibody concentration was determined via titration. Freshly isolated mononuclear cell suspension was centrifuged at 1300 rpm for 10 minutes at room temperature and washed twice with 1 ml of PBS containing 1% BSA ("Sigma-Aldrich", USA). Then, 25 μl of cells (1×10^5) and 1 μl (3 μg) of human IgG (FSUE "NPO "Microgen", Moscow) were added to labeled 12 × 75 mm tubes for Fc receptor blocking. Incubation was performed in the dark at room temperature for 20 minutes. Next, saturating concentrations of anti-TNF RI, anti-TNF RII, anti-IL-1 RI, and anti-IL-1 RII antibodies, as well as 1 μl each of anti-CD3 APC, anti-CD14 FITC, and anti-CD19 PE-Cy7 antibodies for immunophenotyping mononuclear cell subpopulations, were added. The cells with antibodies were incubated for 40 minutes at 4°C in the dark. Subsequently, the cells were washed twice to remove unbound antibodies using 1 ml of PBS with 1% BSA. Then, 100 μl of PBS was added to the tubes, and samples were immediately analyzed on the flow cytometer without fixation.

The investigation of TNF-alpha and IL-1 receptor content was conducted using the same photomultiplier tube (PMT) voltage settings for the PE detector as during the calibration bead analysis. This allowed us to convert fluorescence intensity values into the number of PE molecules per cell. Subsequently, the number of PE molecules per cell was converted into the number of antibody molecules per cell using the known PE-to-antibody molecule ratio of 1:1. Cytometer settings were verified weekly using Cytometer Setup and Tracking (CS&T) beads ("BD Biosciences", USA).

Based on forward and side scatter parameters (on the FSC-A/SSC-A dot plot), analyzed populations located in the lymphocyte and monocyte regions were gated. Then, using subpopulation markers (on APC-A/FITC-A and APC-A/PE-Cy7-A dot plots), subpopulations of T-lymphocytes, B-lymphocytes, and monocytes were gated. At least 10,000 gated events were recorded. An interval gate was set on histograms of the control sample, and subsequently, events positive for membrane-bound receptor markers within the analyzed subpopulations were represented on histograms.

2.3. Fluorescence Reagent Description

The following reagents are being used Table S1:

Characteristic	*Analyte*	*Detector*	*Reporter*	*Manufact.*	*Clone*	*Cat#*
Monocytes	CD14	Anti-CD14	Fitc	eBioscience	61D3	11-0149
B-lymphocytes	CD19	Anti-CD19	*PE-Cy7*	eBioscience	HIB19	25-0199-42
IL-1R1	anti-human IL-1RI	anti-human IL-1RI	PE	R&D Systems	FAB269P	FAB269P
IL-1R2	anti-human IL-1RII	anti-human IL-1RII	PE	R&D Systems	FAB663	FAB663
TNFR1	anti-human TNFR1	anti-human TNFR1	PE	R&D Systems	16803.1	16803.1
TNFR2	anti-human TNFR2	anti-human TNFR2	PE	R&D Systems	22235	22235
T-lymphocytes	CD3	Anti-CD3	APC	eBioscience	OKT3	17-0037

3. Instrument Details

3.1. Instrument Manufacturer

BD Biosciences

http://www.bdbiosciences.com/home/

3.2. Instrument Model

BD FACSAria^{TM} Cell Sorter

Serial number P07900212

3.3. Instrument Configuration and Settings

Flow cytometric analysis was performed using a BD FACSAria flow cytometer (equipped with three lasers—488, 633, and

405 nm) with FACSDiva software version 6.1.2 (BD Biosciences). To check the installation settings, we regularly made performance checks by means of Cytometer Setup and Tracking (CS&T) beads (BD Biosciences).

4. Data Analysis Details

4.1. Compensation Description

Compensation has been performed computationally post-acquisition according to the following spillover matrix (values in %) Table S2:

Experiment Name: Experiment_001
Specimen Name: 07_04
Tube Name: 51_CD

Parameters	Type	Voltage	Log
FSC	A	90	Off
SSC	A	340	Off
FITC	A	494	On
PE	A	440	On
PE-Cy7	A	571	On
APC	A	678	On

Threshold Operator: OR

Threshold Parameters	Threshold
FSC	5,000

Compensation State: Enabled

Fluorochromes	Value(%)
PE - FITC	9.50
PE-Cy7 - FITC	0.30
APC - FITC	0.20
FITC - PE	0.00
PE-Cy7 - PE	0.00
APC - PE	0.00
FITC - PE-Cy7	0.00
PE - PE-Cy7	1.00
APC - PE-Cy7	0.00
FITC - APC	0.00
PE - APC	0.00
PE-Cy7 - APC	0.00

Ratio Parameters	Scaling(%)

4.2. Gating (Data Filtering) Details

4.2.1. The same gating strategy has been used for all data files. All these gates would be reported in a real experiment description. In order to keep this document as a clear and simple example we provide details only on gating of a single list mode data file and we include these as images within this document. Gate Description

The gating strategy involves the following gates:

- FSC-SSC gate to define the leukocytes and monocytes (Figure S3A).
- FSC A -FSC H Exclusion of doublets (Figure S3B).
- APC- PECy7 gate to define the T-leukocytes and B-leukocytes (Figure S3C).
- APC- Fitc gate to define the monocytes (Figure S3D).
- PE gate to define the IL-1R1 on monocytes, T-leukocytes and B-leukocytes (Figure S3E)
- PE gate to define the IL-1R2 on monocytes, T-leukocytes and B-leukocytes (Figure S3F
- PE gate to define the TNFR1 on monocytes, T-leukocytes and B-leukocytes (Figure S4A)
- PE gate to define the TNFR2 on monocytes, T-leukocytes and B-leukocytes (Figure S4B)
-

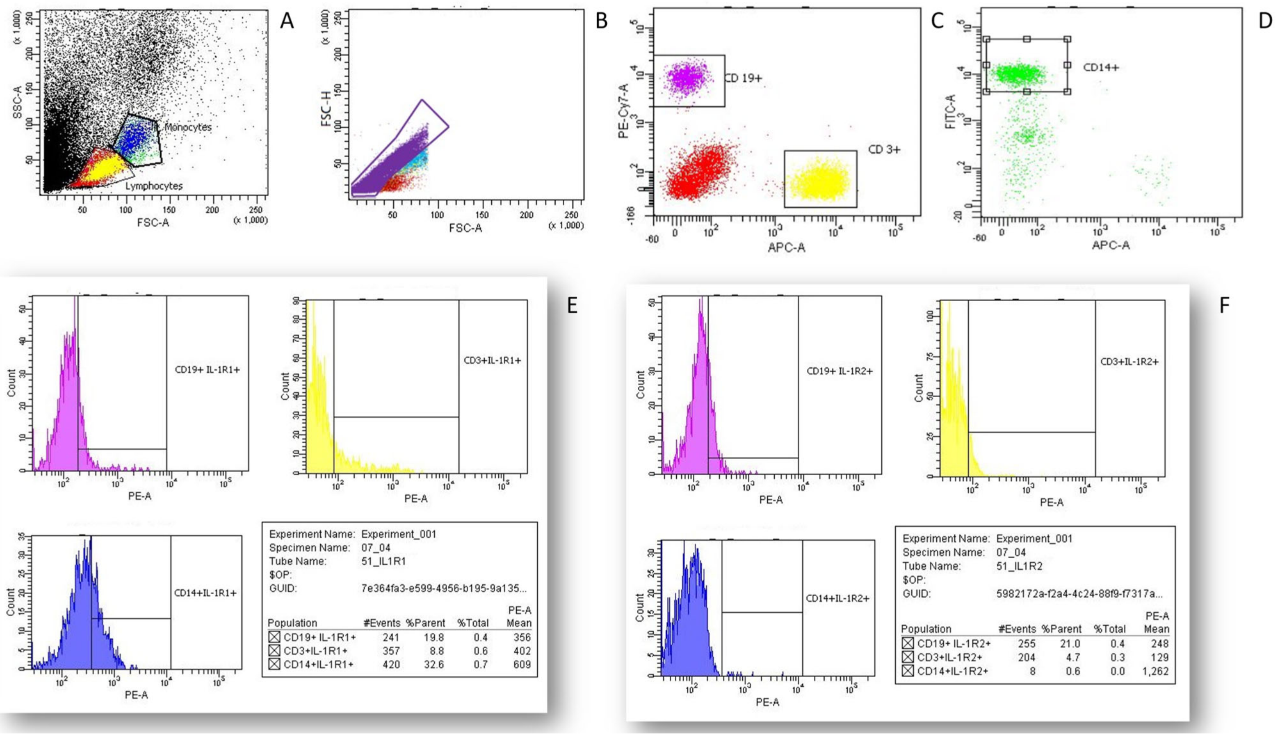

Figure S3. (A-F) gating strategy and ststistical data for monocytes (C), T lymphocytes (C) and B lymphocytes (D), and IL-1R1(E), Il-1R2(F)

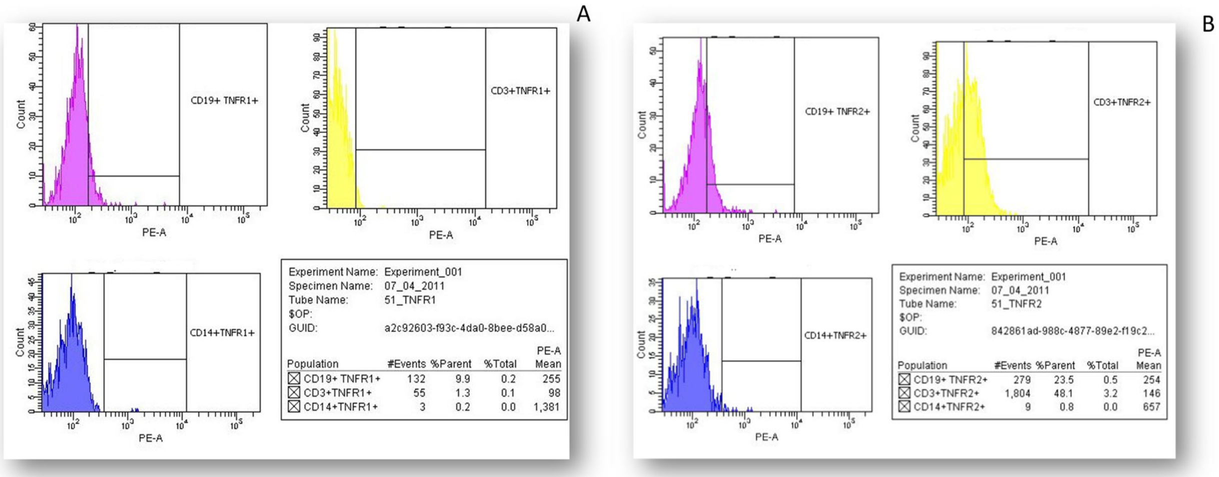

Figure S4. (A B) gating strategy and ststistical data for TNFR1(A), TNFR2(B)
